# Early Non-Invasive Prenatal Testing at 6–9 Weeks of Gestation

**DOI:** 10.3390/genes15070895

**Published:** 2024-07-08

**Authors:** Alexandros Katrachouras, Harry Kontos, Kyriacos Konis, Chara Skentou, George Makrydimas

**Affiliations:** 1 Obstetrics and Gynecology, University Hospital of Ioannina, 455 00 Ioannina, Greece; alexkatra1994@gmail.com (A.K.); haraskentou@gmail.com (C.S.); 2 Department of Molecular Genetics, Genomedica S.A., 185 37 Piraeus, Greece; h.kontos@genomedica.gr; 3 Obstetrics and Gynecology, General Hospital of Arta, 471 00 Arta, Greece; koniskyriacos@gmail.com

**Keywords:** cell-free DNA, fetal screening, low fetal fraction, Vanadis cfDNA test, NIPT

## Abstract

Non-invasive prenatal testing (NIPT) is usually performed beyond 10 weeks of gestation, because earlier in pregnancy, the fetal fraction is low, resulting in failure to obtain reliable results. This study aimed to evaluate the clinical performance of NIPT earlier in pregnancy using a method for cell-free DNA (cfDNA) analysis that eliminates the need for polymerase chain reaction (PCR), DNA sequencing, or microarrays (Vanadis^®^ system, PerkinElmer, Waltham, MA, USA). Cell-free DNA was extracted from the maternal plasma of 30 singleton pregnancies at 6–9 weeks of gestation (group 1) and at 11–14 weeks of gestation of the same patients (group 2). The mean crown-rump length (CRL) and gestational age in group A was 16.12 mm and that in group B was 61.45 mm. In group A, results were obtained in all, but one, cases (97%). From the remaining pregnancies, one miscarried at 8 weeks and, therefore, the follow-up NIPT at 12 weeks could not be performed. The fetal sex was diagnosed correctly in the 28 cases that had a successful early test, and the results were in accordance with the examination at 12 weeks. There were no cases of aneuploidies and disomy was diagnosed correctly in all. The “Vanadis” prenatal NIPT assay can successfully be used early during the first trimester at 6–9 weeks of gestation (early NIPT) to identify the fetal sex. Further studies are needed to explore the diagnostic potential for aneuploidies.

## 1. Introduction

Numerous studies have shown that non-invasive prenatal testing (NIPT) is the most effective method for screening for chromosomal abnormalities; therefore, it is currently used widely in different clinical settings [1,2,3,4,5,6,7,8]. This technique comprises the extraction of fetal cell-free DNA (cfDNA) from maternal plasma and, with the use of several molecular methods, it can detect over 99% of the most common fetal trisomies [1,2,3,4,5,6,7,8]. NIPT is less accurate in detecting other chromosomal aberrations, namely sex chromosome aneuploidies (SCAs) (Klinefelter, Turner, Triple X) or microdeletion syndromes (Cri-du-chat, Prader–Willi, Angelman, DiGeorge, 22q12 deletion, 1p36 deletion) and microduplication syndromes (Beckwith–Wiedemann syndrome, Silver–Russell syndrome) [9,10,11,12,13,14]. Specifically, the larger the fragment, the higher the NIPT detection sensitivity, whereas the sensitivity of copy number variations (CNVs) for fragments <5 M is low with a high missed detection rate [9,10,11,12,13]. Nevertheless, it should be emphasized that in the case of positive results, invasive prenatal diagnosis should be applied for confirmation since the combined false positive rate is about 0.13% for trisomies 13, 18, and 21 and considerably higher for other abnormalities [1,3,4,8].

At present, most commercial NIPT assays use the digital quantification capabilities of advanced sequencing platforms. However, the use of these assays is limited beyond 10 weeks of gestation, because a certain amount of fetal cell-free DNA is required for the successful diagnosis. A low fetal fraction (FF) (<4%) of cfDNA is associated with a high failure rate and a high risk for false negative results [15,16,17,18]. Several factors including maternal body mass index (BMI), twin pregnancy, maternal age, drugs, assisted reproductive technology (ART), and gestational age are considered to affect the fetal fraction of cfDNA [17]. Of those, the latter is proven to be the most important factor [17]. Recent studies have shown that fetal cfDNA can be detected in maternal blood from as early as five weeks of gestation, while its percentage increases with the progress of the gestational age and, at 10 weeks, is about 10% of the total cfDNA, which allows reliable testing and diagnosis [17,19,20,21].

A different method using a high-precision non-next-generation sequencing (NGS) test “Vanadis assay” (PerkinElmer, Waltham, MA, USA) was developed recently. This method is designed for both the diagnosis of fetal sex and the examination of chromosomes 13, 18, and 21 [22,23,24]. It uses a rolling-circle replication-based cfDNA system that screens chromosomes by converting chromosome-unique fragments into digitally quantifiable objects for the evaluation of the common aneuploidies [23,24]. Vanadis achieves high precision by targeting thousands of specific chromosomal sequences, eliminating the need for PCR and performing a high-yield counting of 650,000 molecules per chromosome, which enables the analysis of all samples, including those with low fetal fraction. It could therefore be used earlier in pregnancy when the fetal fraction is low [22,23,24].

The aim of this study is to examine the effectiveness of non-invasive prenatal testing, from as early as six weeks of gestation, using the Vanadis assay.

## 2. Materials and Methods

In Greece, it is a common practice for pregnant women to have an early first trimester ultrasound scan for confirmation of fetal viability, dating of the pregnancy, and diagnosis of multiple and ectopic pregnancies. Women with a viable singleton pregnancy and a measured CRL of 10–21 mm, corresponding to 6–9 weeks of gestation, were offered an early NIPT with the Vanadis system (PerkinElmer, Waltham, MA, USA) (group 1). It was explained that a second blood sample would be collected at the time of the 11–14-week scan as it was calculated by measuring the CRL at the first visit (group 2). It was also mentioned that the results of the first NIPT would not be available for the patients while they would be informed of the results of the second test. Ethical approval for this study was obtained from the ethics committee of the University hospital, Ioannina, Greece (NO 27/27-12-2022). All women were counseled in detail and gave written informed consent.

In women who accepted to participate, the demographics, family, personal, and obstetric history were recorded using the Astraia database. The BMI was calculated. The CRL, gestational, and yolk sac diameters and fetal heart rate were also recorded. The 11–14-week scan was performed according to the Fetal Medicine Foundation (FMF) protocol. All scans were performed by a single operator (GM), certified by the FMF. During the first visit (group1), 10 mL of blood was drawn and collected in a Cell-free DNA BCT^®^ blood collection tube (Streck company, La Vista, NE, USA). These tubes maintain cell-free DNA for up to 14 days at 6 °C to 37 °C and eliminate the need for immediate plasma isolation, easing concerns about pre-analytical variation that arises during shipping and handling. The tube was labeled with a serial number corresponding to each woman and sent the same day to the laboratory. The patient’s demographics and ultrasound data were not accessible by the laboratory staff. The second sample was drawn immediately at the 11–14-week scan (group 2). This sample was appropriately labeled with the name of the pregnant woman and the personal details were given to the laboratory.

The Vanadis NIPT assay was performed according to the manufacturer’s instructions [22]. After successive centrifugations at 1300× *g* for 30 min and 2400× *g* for 10 min, the isolated plasma was placed at −80 °C until the next step of analysis. The cfDNA was extracted from 3 mL of maternal plasma using the Vanadis Extract platform. This was followed by the generation of rolling-circle-labeled replication products (RCPs) on the Vanadis Core platform, which were then imaged and measured with the Vanadis View instrument. Specifically, the extracted cfDNA is first subjected to specific fragmentation using a restriction enzyme. The resulting target cfDNA fragments are similar in size and GC content and are derived from the chromosomes of interest. Probes, designed to hybridize to the target cfDNA fragments to form circular DNA complexes, are mixed with the target cfDNA fragments, backbone oligos, and DNA ligase. By allowing the target cfDNA fragments to hybridize to the probe complex and DNA ligase to seal the nicks, covalently closed circles are generated that each includes a cfDNA target fragment and a corresponding chromosomal tag. All DNA that is not circularized is removed with exonucleases. The DNA circles are copied about 1000 times by rolling-circle amplification (RCA) to generate one rolling circle replication product (RCP), a single stranded concatemer amplification product. The RCPs self-assemble to submicron-sized DNA objects. Because each RCP includes copies of a chromosomal tag, it can be recognized by a corresponding fluorescently labeled oligonucleotide. The labeled RCPs are then deposited to a 96-well nanofilter microplate. The microplate has a nanofilter membrane in the bottom to allow the RCPs to be captured on the plate bottom, while buffer and non-hybridized fluorophores are washed through the membrane. The deposited RCPs are finally imaged and therefore counted through the nanofilter using the Vanadis View imaging instrument [22].

Samples were analyzed in groups of 48 with <50% being the 6–9-week gestation group and the remainder being the 11–14-week gestation controls. The total run time was approximately 48 h per group. Samples were anonymized and they were used in compliance with all applicable laws. This study was conducted single-blindly by identification of the fetuses’ sex in the EARLY NIPT sample which was compared to that (NIPT) at 11–14 weeks of gestation.

## 3. Results

In total, 30 pregnant women agreed to participate in this study (Table 1). The median maternal age was 33 (range: 20–39). The median CRL in group 1 was 16.05 mm (range: 5–29 mm) and the median gestational age was 8.1 weeks (range: 5.8–9.7 weeks). In group 2, the median CRL was 60 mm (range: 49.9–76 mm), while the median gestational age was 12.2 weeks (range: 11.71–13.57). One woman (no 8, Table 1) miscarried at 10 weeks of gestation and the results of the early examination could not be verified. In all, but one (no 6, Table 1) in which the early NIPT failed, the results of the early examination were in accordance with the results of the NIPT at 12 weeks of gestation. There was one case (no 3, Table 1) where the woman decided to undergo amniocentesis due to anxiety, and the karyotype revealed that the fetus was affected by Klinefelter syndrome. This fetus was diagnosed as male in both early and 12-week NIPT. There were no cases of aneuploidies for chromosomes 13, 18, and 21 and disomy was diagnosed correctly in all samples when screened at 6–9 weeks (group 1). Disomy was confirmed for either sample at the NIPT performed at 11–14 weeks (group 2).

## 4. Discussion

The results of this study, involving 30 pregnancies at 6–9 weeks of gestation, demonstrated that the Vanadis prenatal test can be used from as early as six weeks for the determination of fetal sex. Results were obtained in all, but one, cases, excluding one woman that miscarried at 10 weeks, in which the confirmation of the fetal sex was not possible. In the remaining 28 cases, the fetal sex diagnosed early was confirmed at 11^+0^–13^+6^ weeks of gestation. It was also demonstrated that chromosomes 13, 18, and 21 can also be examined successfully in this early stage of pregnancy; however, since there were no cases affected by trisomies in the study group, further studies are needed to prove the efficacy.

Currently, prenatal screening using NIPT is the most effective screening method for common fetal aneuploidies. Compared to the more established combined first trimester screening test (nuchal translucency and biochemical markers), it has the advantage of a higher detection rate, over 99% vs. about 90%, with a very low combined false positive rate of approximately 0.13% vs. 5%, respectively [1,2,3,4,8]. However, this technique is complex to perform with high cost and it is only used widely for patients classified as high risk following the first trimester’s combined test [2]. Cf-DNA testing is also used after parental request; however, most national health systems do not cover the cost.

Additionally, a major limitation for the use of NIPT is that it requires a certain amount of cfFDNA, expressed as the percentage of fetal-to-total DNA fraction (FF), to obtain reliable results [15]. This is because the vast majority of the available NIPT solutions use the power of advanced sequencing platforms or microarray technology [18]. Studies have shown that the lowest FF is considered 4% as, if it is less, the results may become unclear [4,16]. It was reported that at 10 to 11 weeks of gestation, the median percentage of fetal cfDNA is about 10%, and that between 10 and 21 weeks of gestation, the fetal fraction increases 0.10% per week, while after 21 weeks of gestation, it increases at a rate of 1% per week [20]. Maternal factors such as age, weight, race, drugs, ART, autoimmune diseases, and fetal–placental factors such as gestational age, multi-fetal pregnancy, aneuploidies, and certain lab procedures may potentially affect the FF ratio [17]. A recent meta-analysis examining the relation of maternal age, gestational age, maternal weight, and BMI concluded that the only statistically significant association is between gestational age and low FF, emphasizing that the other parameters have no significant relationship with low FF [17]. The authors of this study suggested that NIPT should be offered only beyond 10 weeks of gestation.

NIPT allows the direct screening from maternal plasma for fetal chromosomal abnormalities as early as 10 weeks of gestation [25,26]. The Vanadis system (PerkinElmer, Waltham, MA, USA) has two major advantages. First, this method does not depend on advanced genetic readout systems like sequencing or microarrays and that by eliminating the need for NGS and PCR, it reduces the complexity of the test and therefore both the workflow and cost [22,23,27]. Secondly, by directly targeting fragments of chromosomes using specific probes, it may reduce the amount of fetal DNA needed, providing a result with a lower fetal DNA fraction, while presenting a comparable sensitivity and specificity to traditional NIPT assays [22,24]. Previous studies have shown that Vanadis can be successfully performed in cases with low fetal fraction, typically less than 2%, and provide reliable results [22].

The results of this study have shown that NIPT can be applied for the diagnosis of fetal sex from as early as six weeks of gestation. That is extremely important for pregnancies at risk for severe sex-linked disorders. In these cases, the diagnosis of a female fetus could provide early reassurance and reduce the anxiety of the parents and, in those with male fetuses, allow the choice to perform invasive prenatal diagnosis much earlier. Celocentesis, which involves the transvaginal insertion of a needle into the celomic cavity and aspiration of celomic fluid, can be used from 6 weeks of gestation and provide reliable results [28,29,30]. In our recent study of 402 pregnancies undergoing celocentesis, we have shown that celomic fluid was successfully aspirated in 99.8% cases and that the vast majority of women had no or only mild discomfort. The use of specific antibodies for the positive selection of fetal cells and the use of a micromanipulator to pick up individual cells resulted in obtaining reliable results following celocentesis. Finally, the fetal loss rate was 2.3%, which is remarkable in this early stage of pregnancy [30]. Therefore, celocentesis could also be performed for the confirmation of pathological results following the early NIPT (e.g., an aneuploidy) the same way chorionic villus sampling is used, if the abnormal NIPT results are provided beyond 10 weeks of gestation.

## 5. Limitations

The main limitation of our study is the relatively small number of cases included. Furthermore, in the study population, no chromosomal abnormality was detected; therefore, the ability of diagnosing aneuploidies was not verified. However, in 97% of the cases, the test provided results.

## 6. Future Work

In order to more accurately validate “Early NIPT” at 6–9 weeks of gestation, we are designing a much larger double-blind study, employing 1000 patients for testing both at 7 and 12 weeks.

## 7. Conclusions

The “Vanadis” prenatal NIPT assay can successfully be used early during the first trimester at 6–9 weeks in order to identify the fetal sex.

## Figures and Tables

**Table 1:** Characteristics, measurements, and results between the two groups. * An amniocentesis was performed due to maternal anxiety. The fetus’s karyotype revealed an additional X chromosome (XXY)—Klinefelter syndrome. Brackets point out the origin of either sample: (1) = NIPT at 6–9 weeks—group 1; (2) = NIPT at 11–14 weeks—group 2. Abbreviations used in table: Mat.Age = maternal age, BMI = body mass index, CRL = crown rump length, GA = gestational age.

NO	Mat.Age	BMI _(1)_	CRL (mm) _(1)_	GA _(1)_	SEX _(1)_	BMI _(2)_	CRL (mm) _(2)_	GA _(2)_	SEX _(2)_
1	27	25.8	13.4	7.85	FEMALE	23.6	71.0	13.28	FEMALE
2	39	21	12.9	7.71	FEMALE	22.1	52.0	11.71	FEMALE
3	32	19.3	23.4	8.71	MALE	21.3	70.3	12.71	MALE *
4	33	21.5	14.3	7.71	MALE	22.5	57.4	12.28	MALE
5	36	24.5	18.2	8.14	FEMALE	25.2	55.0	12.14	FEMALE
6	37	22.1	12.7	7.14	FAILURE	22.1	65.6	12.85	MALE
7	33	19.5	19.9	8.71	MALE	20.9	51.7	11.85	MALE
8	30	21.8	17.0	8.28	MALE	22	MISCARRIAGE		
9	26	20.3	11.8	7.71	FEMALE	19.8	52.1	11.85	FEMALE
10	38	27.5	19.9	8.71	MALE	27.9	76.0	13.57	MALE
11	38	24.4	19.1	8.28	MALE	24.6	75.1	13.57	MALE
12	30	21.2	15.1	8.28	FEMALE	20.9	71.8	12.28	FEMALE
13	33	25.3	17.3	8.14	MALE	26	70.4	13.14	MALE
14	38	31.2	16.9	8.28	MALE	32	60.0	12.28	MALE
15	35	27.5	16.1	7.85	FEMALE	27.9	55.8	12.0	FEMALE
16	29	22.7	16.0	8.00	FEMALE	23.3	68.3	13.0	FEMALE
17	37	21.1	14.0	7.71	MALE	22	55.8	12.14	MALE
18	35	21	16.0	8.14	FEMALE	21.8	55.2	12.14	FEMALE
19	29	29.1	21.0	8.71	FEMALE	30.1	54.6	11.85	FEMALE
20	26	26	19.0	8.42	FEMALE	26	64.5	12.85	FEMALE
21	29	22.5	20.0	8.57	FEMALE	23.6	49.9	11.71	FEMALE
22	27	33.2	11.6	7.57	FEMALE	33.6	59.2	12.0	FEMALE
23	20	25.3	05.0	5.85	MALE	26.3	70.2	13.14	MALE
24	30	32	20.6	9.0	FEMALE	29.7	57.7	12.28	FEMALE
25	34	20.9	23.8	8.57	FEMALE	20.9	56.9	11.71	FEMALE
26	33	32.4	10.0	7.0	MALE	32.6	59.2	12.57	MALE
27	37	25.5	16.0	8.14	MALE	23.6	60.0	12.57	MALE
28	37	22.3	29.0	9.71	MALE	22.3	60.9	12.57	MALE
29	28	22.8	15.0	8.14	MALE	23.1	60.7	12.57	MALE
30	29	22.6	10.5	7.14	MALE	22.1	64.9	12.71	MALE

## Data Availability

Data of our study are fully provided in Table 1. No additional supporting data is available.
